# Information maximizing component analysis of left ventricular remodeling due to myocardial infarction

**DOI:** 10.1186/s12967-015-0709-4

**Published:** 2015-11-03

**Authors:** Xingyu Zhang, Bharath Ambale-Venkatesh, David A. Bluemke, Brett R. Cowan, J. Paul Finn, Alan H. Kadish, Daniel C. Lee, Joao A. C. Lima, William G. Hundley, Avan Suinesiaputra, Alistair A. Young, Pau Medrano-Gracia

**Affiliations:** Department of Anatomy with Radiology, Grafton Campus, University of Auckland, 85 Park Road, Grafton, Auckland, 1148 New Zealand; The Donald W. Reynolds Cardiovascular Clinical Research Center, The Johns Hopkins University, Baltimore, USA; National Institute of Biomedical Imaging and Bioengineering, Bethesda, MD USA; Department of Radiology, UCLA, Los Angeles, USA; Feinberg Cardiovascular Research Institute, Northwestern University Feinberg School of Medicine, Chicago, USA; Section of Cardiology Medical Center Blvd, Winston Salem, NC USA

**Keywords:** Cardiac remodeling, Information maximizing component analysis, Magnetic resonance imaging, Linear discriminant analysis, Logistic regression

## Abstract

**Background:**

Although adverse left ventricular shape changes (remodeling) after myocardial infarction (MI) are predictive of morbidity and mortality, current clinical assessment is limited to simple mass and volume measures, or dimension ratios such as length to width ratio. We hypothesized that information maximizing component analysis (IMCA), a supervised feature extraction method, can provide more efficient and sensitive indices of overall remodeling.

**Methods:**

IMCA was compared to linear discriminant analysis (LDA), both supervised methods, to extract the most discriminatory global shape changes associated with remodeling after MI. Finite element shape models from 300 patients with myocardial infarction from the DETERMINE study (age 31–86, mean age 63, 20 % women) were compared with 1991 asymptomatic cases from the MESA study (age 44–84, mean age 62, 52 % women) available from the Cardiac Atlas Project. IMCA and LDA were each used to identify a single mode of global remodeling best discriminating the two groups. Logistic regression was employed to determine the association between the remodeling index and MI. Goodness-of-fit results were compared against a baseline logistic model comprising standard clinical indices.

**Results:**

A single IMCA mode simultaneously describing end-diastolic and end-systolic shapes achieved best results (lowest Deviance, Akaike information criterion and Bayesian information criterion, and the largest area under the receiver-operating-characteristic curve). This mode provided a continuous scale where remodeling can be quantified and visualized, showing that MI patients tend to present larger size and more spherical shape, more bulging of the apex, and thinner wall thickness.

**Conclusions:**

IMCA enables better characterization of global remodeling than LDA, and can be used to quantify progression of disease and the effect of treatment. These data and results are available from the Cardiac Atlas Project (http://www.cardiacatlas.org).

## Introduction

### Background

Changes in the geometry of the left ventricle (LV) of the heart typically occur after myocardial infarction (MI) in response to disease processes; this phenomenon is clinically termed *remodeling* [1–3]. Important diagnostic information can be obtained from the degree and pattern of remodeling in the ischemic heart [4, 5]. For example, remodeling associated with increased heart size is predictive of poor outcomes [5], while sphericalization of the LV has been linked with increased mortality [4]. The relationship between end-systolic volume and end-diastolic volume can distinguish patient phenotypes [6]. However, traditional clinical indices currently used to quantify remodeling are limited to simple measures of mass and volume, or ventricular dimension ratios, discarding much of the available shape information.

Several prospective large-scale population-based studies have included cardiovascular magnetic resonance (CMR) imaging as part of their assessment [1, 7, 8], collecting phenotypic data on cardiac disease. CMR, as a non-invasive radiation-free modality, provides rich and detailed quantitative data of the heart function and structure. Non-invasive tomographic imaging in combination with shape analysis is leading to an increasing number of applications exploiting these data through statistical analysis of cardiac shape and motion [9]. In particular, finite-element model analysis has been applied to model LV shape and function, providing accurate and reproducible customization of a model template to each patient with minimal user interaction [10–12].

### Related work

Principal component analysis (PCA) has been extensively used to analyze shape patterns found in population groups. PCA has been applied to analyze heart shape [13] and motion [14], aid in 3D segmentation [15], and cluster shape variation [16, 17]. In our previous work, PCA scores were used to characterize remodeling due to MI [17]. However, PCA is an unsupervised feature extraction method that does not always result in clinically interpretable features. Typically, many PCA scores are required to achieve discriminatory power [18–20]. This has led researchers to investigate supervised feature extraction techniques to generate more powerful and efficient shape indices. Linear discriminant analysis (LDA) is a commonly used supervised feature extraction technique for classification problems [21], and has been widely applied in image processing areas [22] including characterization of cardiac disease in limited datasets including endocardial information only [23]. However, LDA relies on the assumptions of Gaussian class distributions and homoscedasticity. Information maximizing component analysis (IMCA) is an extension of LDA developed by Carter et al. [24], which does not rely on these assumptions. An unsupervised version of the method was applied to flow cytometry analysis, requiring fewer modes than PCA and providing better disease classification [18]. A supervised version has been applied to satellite image high dimensional data [25]. However, the performance of this method in cardiac remodeling has not been investigated.

Previous methods applied to cardiac disease have included support vector machines [26], neural networks [27] and Shannon’s differential entropy [28]. However, the number of cases has been limited and most methods do not have a theoretical basis in statistical theory. Since IMCA extends LDA to applications where the underlying assumptions of LDA are violated, it is reasonable to hypothesize that IMCA will outperform LDA in this context. The contributions of this paper are therefore (1) the application of supervised feature extraction algorithms to the largest dataset of both normal and MI patients currently available, and (2) the comparison of IMCA with LDA for the quantification of remodeling due to cardiac disease. We used logistic regression (LR) to assess the relationship between the presence of MI and the remodeling indices derived from LDA and IMCA and establish a classification model. Goodness-of-fit performance measures were then used to rank the discriminatory power of the remodeling indices.

## Data and methods

### Participants

LV shape models were obtained from the Cardiac Atlas Project, a resource for large scale cardiac image analysis and computational anatomy [29] (http://www.cardiacatlas.org). We compared shape models derived from 300 MI patients with 1991 asymptomatic volunteers. Models for MI patients were derived from images contributed from the baseline imaging examination of the Defibrillators to Reduce Risk by Magnetic Resonance Imaging Evaluation (DETERMINE) study, which studied patients with coronary artery disease and mild to moderate LV dysfunction [30]. Models for asymptomatic volunteers were derived from images contributed from the baseline imaging examination of the Multi Ethnic Study of Atherosclerosis (MESA) [8], comprising volunteers with no clinical evidence of disease (although sub-clinical disease may have been present). Details of the exclusion and inclusion criteria, imaging protocols, and correction of shape bias between imaging protocols have been described elsewhere [17, 31]. Participant characteristics in the two groups were significantly different in many demographic parameters (Table 1). The DETERMINE group was more predominantly male, older, taller, heavier, had higher diastolic blood pressure, less history of diabetes and bigger volume than MESA participants. Variables including gender, age, height, weight, blood pressure, diabetes history and smoking status were therefore included in the LR models as baseline variables to calculate the odds ratio of the derived remodeling indices without the influence of confounding factors.

**Table 1:** Demographics for the MESA and DETERMINE datasets (mean ± SD)

	Units	DETERMINE	MESA
Sex (female/male)^‡^		60/238	1034/975
Age^†^	years	62.76 ± 10.80	61.47 ± 10.15
Height^‡^	cm	173.91 ± 9.80	165.97 ± 9.99
Weight^†^	kg	90.06 ± 19.15	76.75 ± 16.50
Systolic BP	mmHg	127.50 ± 20.14	126.00 ± 22.00
Diastolic BP^‡^	mmHg	73.86 ± 11.34	71.49 ± 10.33
Diabetes history^‡^	%	13.11	35.67
Smoking status	%	12.51	11.33
ESVI^‡^	ml/m^2^	58.36 ± 24.39	25.48 ± 8.69
EDVI^‡^	ml/m^2^	96.53 ± 25.03	67.83 ± 13.29

For continuous variables, p values report a Wilcoxon signed-rank test of the null hypothesis. For categorical variables the p value reports a *χ* ^2^ test of the null hypothesis

*BP* blood pressure, *ESVI* end-systolic volume index, *EDVI* end-diastolic volume index

^†^p < 0.05; ^‡^p < 0.01

### Study design

**Fig. 1:**
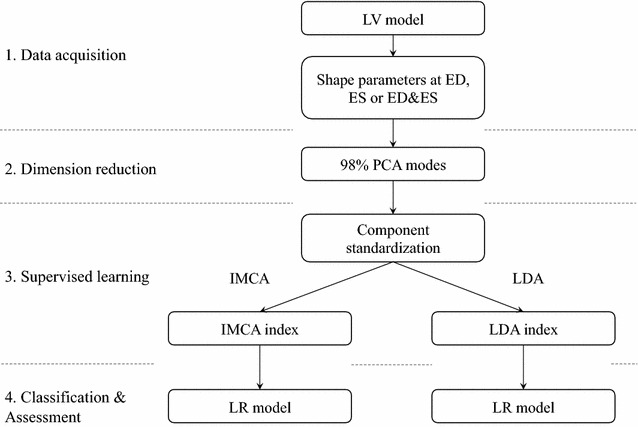
Data processing flow chart. Firstly, PCA was performed on the shape parameters of left ventricle finite element model (LV FEM) at end diastole (ED), end systole (ES), and using the combination of ED and ES. Secondly, PCA modes which accounted for 98.5 % of the total variation were standardized. Thirdly, LDA and IMCA were applied on the standardized components to generate global modes of variation which were assessed with a logistic regression classification model (LR model), including other confounding variables

Data were analyzed following the flow chart in Fig. 1. Finite-element models were customized to the MRI scans points at end-diastole (ED) and end-systole (ES). A set of evenly spaced homologous points were generated on the ventricular surfaces by subdivision, resulting in 1682 Cartesian $(x_{i} ,y_{i} ,z_{i} )$ points per case in atlas coordinates, which served as shape parameters or image-derived features. The point sets from each model were rigidly aligned with the mean using the Procrustes alignment method [32]. Since heart size is an important clinical indicator of disease, scale variations were not removed. Principal component analysis was applied to reduce the dimensionality of the shape space but still retain 98 % of the population variation. IMCA and LDA were performed on the standardized PCA scores. These generated scalar indices associated with global remodeling due to MI. Finally, a LR model was used to analyze the ability of the remodeling indices to characterize MI patients. Three types of shape analysis were considered: (1) the ED frame only; (2) the ES frame only; (3) a combination of ED and ES frames. For the latter the sampled points for both frames were concatenated into a single shape vector for each case.

### Principal component analysis

Currently, principal component analysis [33] is widely used to reduce the number of variables (dimension reduction) while retaining most of the variation in a coherent dataset. Using consecutive orthogonal rotations, PCA projects the data onto a linear space of maximum-variance directions but reduced dimension, generated by eigenvectors or *modes*. In this work, principal component analysis was used as a preliminary dimension reduction step, to ensure convergence of the IMCA algorithm. Enough PCA modes to explain 98.5 % of the total variance were retained.

### Linear discriminant analysis

LDA, or Fisher’s linear discriminant, calculates a new variable that is a linear combination of the original predictors, by maximizing the differences between the predefined groups. In contrast to PCA, LDA considers class membership for dimension reduction. This can be viewed as a stringent dimension reduction technique that compresses the *p*-dimensional predictors into a one-dimensional line. Mathematically, LDA tries to find the projection matrix which maximizes the between-class scatter matrix and minimizes the within-class scatter matrix of projected points. The key idea of LDA is to separate the class means of the projected samples while achieving a small variance around these means. The derived features of LDA can be shown in the form of:

1 $$D = w_{1} X_{1} + w_{2} X_{2} + \cdots + w_{m} X_{m}$$

where *D* is the discriminant score which is a weighted linear combination of the *m* predictors. The weights are estimated to maximize the differences between class mean discriminant scores. Generally, those predictors which have large dissimilarities between class means will have larger weights, at the same time weights will be small when predictor class means are similar. Note that LDA assumes that the conditional probabilities of each class are normally distributed and that the class covariances are equal (homoscedasticity).

### Information maximizing component analysis

IMCA models each class as a probability density function (PDF) on a statistical manifold which can be projected into a low dimensional Euclidean space [18]. The Fisher information distance between PDFs is used to describe the similarity between classes. The Fisher information distance between two distributions $p(x;\theta_1)$ and $p(x;\theta_2)$ is defined by:

2 $$DF(\theta_1,\theta_2) = \mathop {\hbox{min} }\limits_{\theta :\theta (0) = \theta 1,\theta (1) = \theta 2} \int {\sqrt {\left(\frac{d\theta }{dt}\right)^{T} [I(\theta )]\left(\frac{d\theta }{dt}\right)dt} }$$

where $\theta_1$ and $\theta_2$ are the parameters corresponding to the two PDFs, $\theta (t)$ is the parameter path along the manifold and $I(\theta )$ is the Fisher information matrix whose elements are defined as:

3 $$[I(\theta )] = \int {f(X;q)} \frac{\partial \log f(X;\theta )}{{\partial \theta^{i} }}\frac{\partial \log f(X;\theta )}{{\partial \theta^{j} }}dX$$

While the Fisher information distance cannot be exactly computed without knowing the parameterization of the manifold, it can be approximated by the Kullback–Leibler divergence [25], denoted $D_{KL} (p_{i} ,p_{j} )$.

The IMCA projection is defined as one that maximizes the Fisher information distance between classes. Specifically, let $\chi = \left\{ {X_{1} ,X_{2} } \right\}$ be a family of data sets where $X_{1}$ corresponds to samples from MESA and $X_{2}$ corresponds to samples from DETERMINE, estimating the PDF of $X_{i}$ as $p_{i}$. Following [17], we refer to $D_{KL} (p_{i} ,p_{j} )$ as $D_{KL} (X_{i} ,X_{j} )$ with the knowledge that the divergence is calculated with respect to PDFs, not realizations. We wish to find a single orthonormal projection matrix A such that

4 $$A = \arg \mathop {\hbox{max} }\limits_{{A:A^{T} A = I}} \left\| {D_{KL} (AX_{i} ,AX_{j} )} \right\|_{F}$$

where $I$ is the identity matrix and $D_{KL}$ is the 2 × 2 matrix of Kullback–Leibler divergences.

We used the Gradient Descent algorithm to find the optimal solution. IMCA can be viewed as a generalized and orthogonal version of LDA, which does not make assumptions on the class distributions [24].

### Logistic regression statistics

LR models [34] were used to quantify the ability of the remodeling indices to characterize MI patients. LR is a statistical classification model, based on probabilistic theory, and is typically used to predict a binary response from continuous, binary, or canonical variables. In the current study, MESA cases (non-patients) were assigned a 0-label whereas DETERMINE cases (patients) were assigned a 1-label, indicating disease. Prediction power after adjustments for age, sex, height, weight, systolic blood pressure, diastolic blood pressure, smoking status and diabetes status were assessed, and the regression coefficient (*β*_*1*_) for each mode was calculated from the multivariable logistic models. Age, sex, height, weight, systolic blood pressure, diastolic blood pressure, smoking status and history of diabetes were used to develop the baseline model. These variables were also included in all the models since these variables can be confounding factors between the disease and shape features. Goodness-of-fit measures of each LR model were examined to determine how well the regression model distinguishes between non-patients and patients. Three common statistics used to quantify the goodness-of-fit of this type of classification models are deviance, Akaike information criterion (AIC) and Bayesian information criterion (BIC) [35, 36]:

5 $$\begin{aligned} Deviance &= - 2\log (L) \hfill \\ AIC &= - 2\log (L) + 2k \hfill \\ BIC &= - 2\log (L) + k*{\text{log}}(n) \hfill \\ \end{aligned}$$

where the *L* represents the log-likelihood of the model, *k* is the number of estimated parameters and *n* is the sample size. In all three measures, a lower number is indicative of a better model. The areas under the curve (AUC) of the receiver operating characteristic (ROC) curves were also computed and compared using the non-parametric method introduced in [37].

## Results

**Fig. 2:**
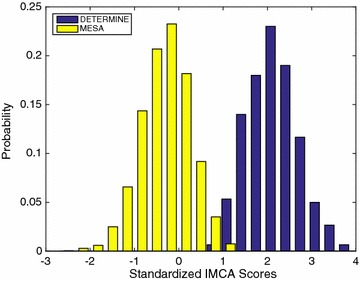
Distribution of IMCA Scores of MESA and DETERMINE for the best case (ED&ES). ED and ES figures do not show perceivable differences in their equivalent plots and are therefore omitted

**Fig. 3:**
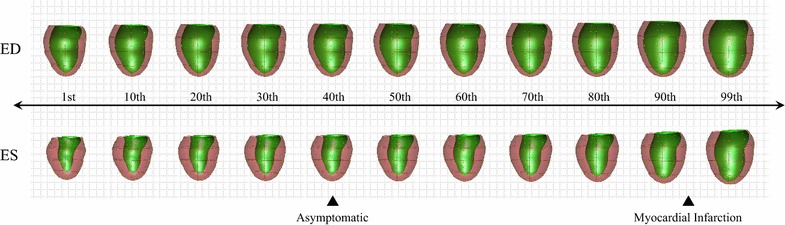
The derived shape indices allow for a continuous representation of disease remodeling. In the *figur*e, the corresponding shapes from the percentiles of the IMCA ED&ES index are shown. Mean values (*black triangles*) for the asymptomatic (MESA) and myocardial infarct group (DETERMINE) show over 50 percentiles of separation for this index. Percentiles correspond to the histogram shown in Fig. 2

PCA modes accounting for 98.5 % of the total variance at ED and ES as well as their combination (ED&ES) led to 55 PCA modes for ED, 50 for ES, and 92 for (ED&ES). IMCA and LDA were performed on the standardized PCA scores, leading to a single remodeling score per case. The standardized LDA and IMCA scores are shown in Table 2. All the scores between MESA and DETERMINE were significantly different (p < 0.0001). The distribution of IMCA scores at ED&ES between MESA and DETERMINE is shown in Fig. 2. The asymptomatic group and the myocardial infarction group were best discriminated with IMCA scores. The Pearson correlation coefficients among the estimated modes are given in Table 3. All the IMCA and LDA modes were highly correlated. This indicates that the remodeling modes obtained with the two methods were strongly related between IMCA and LDA, and between the ED, ES and the ED&ES atlases. The mode of shape variation associated with both IMCA and LDA methods was visualized by combining the PCA shape modes with the optimized weights found in each method. Figure 3 shows how these new indices of global remodeling create a continuum where cases can be scored according to their degree of severity; in particular, it shows that the IMCA ED&ES mode captures the larger size and more spherical shape, bulging of the apex, and thinner wall thickness, which are known clinically to be associated with remodeling after myocardial infarction. The mode shapes derived from all IMCA and LDA modes were visually similar and are therefore not shown. In the experiments, IMCA required 8.13 s processing compared with 0.75 s for LDA on a standard desktop (Intel i5 quad-processor 3.4 GHz, 8 GB RAM).

**Table 2:** LDA and IMCA Scores for MESA and DETERMINE (mean ± SD)

	MESA	DETERMINE	p value
ED LDA	−0.30 ± 0.61	1.99 ± 0.77	<0.0001
ES LDA	−0.33 ± 0.48	2.18 ± 0.80	<0.0001
ED&ES LDA	−0.34 ± 0.44	2.25 ± 0.73	<0.0001
ED IMCA	−0.29 ± 0.66	1.94 ± 0.65	<0.0001
ES IMCA	−0.31 ± 0.58	2.07 ± 0.68	<0.0001
ED&ES IMCA	−0.32 ± 0.56	2.13 ± 0.57	<0.0001

**Table 3:** Correlation coefficients among IMCA and LDA modes

	ED IMCA	ES IMCA	ED&ES IMCA	ED LDA	ES LDA	ED&ES LDA
ED IMCA	1.00					
ES IMCA	0.81	1.00				
ED&ES IMCA	0.87	0.92	1.00			
ED LDA	0.97	0.82	0.86	1.00		
ES LDA	0.80	0.95	0.90	0.83	1.00	
ED&ES LDA	0.86	0.92	0.95	0.88	0.97	1.00

All the correlation coefficients are statistically significant p < 0.05

**Fig. 4:**
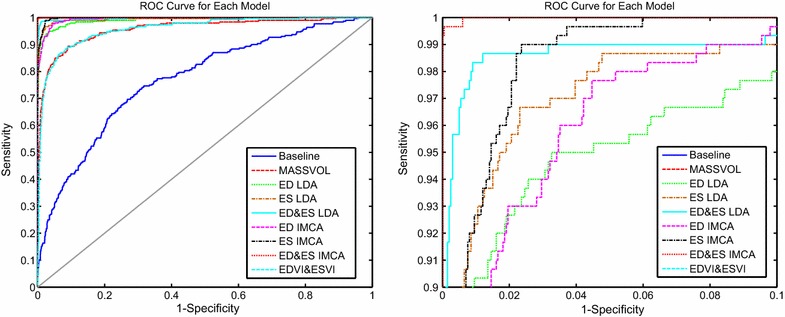
ROC curves for the analyzed logistic regression models. *Right figure* zooms into the *upper-left* corner

Nine logistic regression models were studied (Table 4; Fig. 4). The baseline model included only the sex, age, height, weight, diastolic blood pressure and history of diabetes. The MASSVOL model include baseline variables as well as ED volume, ES volume and LV mass since these are the standard remodeling indices currently used clinically [17]. Also, for comparison with [6], an ESVI+EDVI model was formulated to include ES volume index and ED volume index (together with baseline variables). IMCA and LDA models included the baseline variables plus the single standardized index derived from IMCA or LDA respectively. Both IMCA and LDA modes showed very high odds ratio of the disease (all ORs were over 100). All goodness-of-fit measures (Deviance, AIC, BIC and AUC) of the IMCA and LDA models were smaller than the baseline model and the MASSVOL model. ES shape feature models showed better performance than the analogous ED shape feature models for both IMCA and LDA. The combination of ED&ES shape features also improved agreement over just ES or ED shape features separately. Finally, the combined ED&ES IMCA logistic model achieved the lowest Deviance, AIC, BIC and highest AUC.

**Table 4:** Assessment table showing measures of goodness-of-fit for the eight logistic regression models

	LR coefficient (*β*_*1*_)	σ (*β*_*1*_ *)*	P value	Deviance	AIC	BIC	AUC (%)
Baseline	–	–	–	1500	1518	1569	76.94
MASSVOL + Baseline	–	–	–	719	743	812	95.70
EDVI + ESVI + Baseline	–	–	–	751	773	837	95.89
ED LDA Score + Baseline	5.1651	0.3736	<0.0001	307	327	385	99.15
ES LDA Score + Baseline	4.8458	0.3724	<0.0001	241	261	319	99.42
ED&ES LDA Score + Baseline	7.0549	0.7585	<0.0001	130	150	207	99.77
ED IMCA Score + Baseline	6.1631	0.4974	<0.0001	271	291	348	99.49
ES IMCA Score + Baseline	6.9857	0.6593	<0.0001	179	199	256	99.81
ED&ES IMCA Score + Baseline	37.1034	13.5261	0.0061	16	36	93	99.99

Coefficients show the differential weight when compared to the Baseline model

Considering the AUC as a measure of discriminatory power, all LDA and IMCA modes had significantly more discrimination than the baseline (p < 0.05) and MASSVOL models (p < 0.05). Both the LDA and IMCA ED&ES coupled modes showed better discrimination than either the ED and ES modes (p < 0.05). The IMCA ED&ES and IMCA ED showed better discrimination than their corresponding LDA modes (p < 0.05), but the difference between the IMCA ES mode and the LDA ES mode was not significant (p > 0.05). In addition, the LDA assumption of normality within each class was examined using the method described in [38], and the class covariance equality assumption was tested using Bartlett’s modification of the likelihood ratio test [39]. Both assumptions were found to be violated (p < 0.05 for each).

## Discussion

Patients with myocardial infarction undergo significant shape changes due to cardiac remodeling. Previously, unsupervised dimension reduction methods have shown superior performance to traditional mass and volume analysis in large data sets [17]. In the current paper, we explored more effective indices of cardiac remodeling using supervised feature extraction methods and compared IMCA with LDA in a large dataset.

To our knowledge, this is the first time that supervised feature extraction has been used in a large CMR dataset, and that IMCA has been applied in this context, compared with LDA. The advantage of the supervised techniques developed in this work is that a single remodeling index is found, as opposed to many remodeling indices for unsupervised PCA logistic models (in [17] we used 13-20 PCA modes describing 90 % of the total variance), and this single remodeling index derived from IMCA or LDA can efficiently quantify the main shape difference between the patients and asymptomatic volunteers. Since these global shape indices define a direction in shape space, this method can also be used as a clinical tool to characterize the patterns of change due to remodeling. By projecting the IMCA modes back onto the population space (Fig. 3), we can visualize the shape changes due to MI remodeling, such as the increase in size of the LV, and the decrease in wall thickness. This mode can be used for tracking individual patients over time future studies, by quantifying the degree to which their LV shapes compare with the remodeling spectrum. This method can be generalized to any disease group, although we only applied the method to patients with myocardial infarction in this study.

Compared to PCA, IMCA and LDA are supervised feature extraction methods, which can result in fewer modes to characterize the remodeling. Thus, a single IMCA or LDA mode obtained better classification results than using 10 PCA modes in our previous study [17]. This indicates that IMCA and LDA can effectively characterize shape variation due to remodeling with a single number. This number captures variations due to size, sphericity and wall thickness (Fig. 3), which are common across a number of different patient infarct locations. Although myocardial infarction is a regional disease, the IMCA mode extracts a global remodeling index which is indicative of a global physiological response to this localized insult.

We also found that the IMCA modes and LDA modes were highly linearly correlated, which shows that the modes characterizing the two groups are statistically dependent across ED, ES and the combination of ED and ES. The combination of ED&ES shape features extracted by IMCA was better at discriminating disease than IMCA ES shape features models, and the IMCA ES index was better than the corresponding ED index. This indicates that the shape either at ED or at ES contains unique clinical information and their combination contains more. Notice that derived measures such as motion ED-ES or additional geometric features such as curvature are indirectly included since these can be derived from the analyzed parameters.

**Fig. 5:**
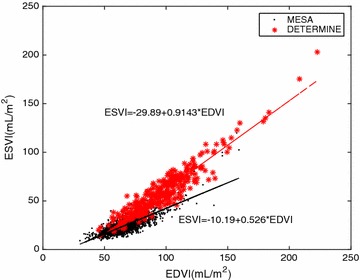
Graph of ESVI versus EDVI with linear regression lines for MESA (control group) and DETERMINE (MI patients)

**Fig. 6:**
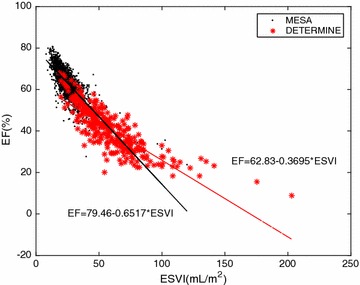
Graph of EF versus ESVI with linear regression lines for MESA (control group) and DETERMINE (MI patients)

Several groups have previously demonstrated the importance of relationships between EF and ES volume, or ES volume and ED volume, in the discrimination between patient groups. White et al. [5] found two distinct regression lines for MI patient groups with different prognosis. Kerkhof et al. [6] extended this concept to plot ES volume against ED volume (each indexed by body surface area), showing discrimination between patients with preserved and reduced EF. A similar analysis in the current cohort showed that the slope of the ES volume to ED volume relationship was significantly higher (p < 0.001) for MI patients than asymptomatic controls (Fig. 5). The derived EF to ES volume relationships are shown in Fig. 6 (p < 0.001 for difference between slopes). These data suggest that linear regression models which include ES and ED volume will perform well for MI patients, a prediction which is confirmed by the high area under the ROC curve for the MASSVOL and the ESVI+EDVI logistic regression models (Table 4).

IMCA is based on information theory, the goal of which is to maximize the information separation between the groups. IMCA methods can generate more than one orthogonal mode, depending on the dimension of the information present in the class distributions. We also calculated the second and third (orthogonal) IMCA modes, but these performed similarly to the single mode analysis and added no more discriminatory power to the classification model.

Limitations of this study include the different source of the two groups (MESA and DETERMINE) and the requirement for correction of the MESA shape models to control for bias between different imaging protocols. The transformation from GRE to SSFP models was learned using 40 normal volunteers. Shape bias arising from these protocol differences may still be present. While [31] showed that this was sufficient to robustly characterize the transformation, more cases would provide a greater variation of heart shape and might improve the transformation parameters. Feature extraction techniques typically rely on data-derived information only and do not consider other clinical data such as sex, age or BMI. Future feature extraction techniques targeting specific subgroups could be performed. Methods to decompose the deformation of the left ventricle between ED and ES into separate deformation modes such as longitudinal shortening, wall thickening, and twisting were developed in previous studies [40].

## Conclusion

Both LDA and IMCA performed well in our experiments and derived similar shape modes. Both performed better than all traditional indices. IMCA had better discriminatory power in ED and ED&ES data than LDA, possibly because the data violated the LDA underlying assumptions.

These synthetic clinically motivated modes may be used to quantify the ventricular remodeling in the future. Although feature extraction techniques such as PCA, IMCA or LDA can extract the main features from the ventricular shape parameters, these techniques are all data-driven methods, which means that the modes extracted from these methods change with the data. However in this research the large number of cases ensures a more robust result from a population perspective.

In conclusion, a single remodeling index derived from IMCA analysis of ED and ES shapes was found to discriminate patients and asymptomatic volunteers with an accuracy of 99 %. The data and results are available from the Cardiac Atlas Project (http://www.cardiacatlas.org).
